# Marijuana Use and Hypothalamic-Pituitary-Adrenal Axis Functioning in Humans

**DOI:** 10.3389/fpsyt.2018.00472

**Published:** 2018-10-01

**Authors:** Anita Cservenka, Sarah Lahanas, Julieanne Dotson-Bossert

**Affiliations:** School of Psychological Science, Oregon State University, Corvallis, OR, United States

**Keywords:** marijuana, hypothalamic-pituitary-adrenal axis, cortisol, adrenocorticotropic hormone, tetrahydrocannabinol

## Abstract

Preclinical studies suggest cannabinoids affect functioning of the hypothalamic-pituitary-adrenal (HPA) axis, but little is known about the effects of marijuana (MJ) use on HPA axis functioning in humans. Since previous work indicates substances of abuse may dysregulate the HPA axis, it is critical to understand how MJ use affects HPA axis activity. Here, we review studies that (a) examined the effects of acute MJ administration on HPA axis functioning, (b) investigated the impact of stress on HPA axis functioning in MJ users, (c) examined the effect of chronic MJ use on basal cortisol levels, and (d) studied the relationship between MJ use and the cortisol awakening response (CAR). Findings indicate acute MJ administration typically raises cortisol levels, but this increase is blunted in MJ-dependent users relative to controls. Frequent MJ users have blunted adrenocorticotropic hormone and cortisol reactivity in response to acute stress. These findings suggest HPA axis activity may be dysregulated by heavy MJ use. Alternatively, dysregulation of the HPA axis may be a risk marker for heavy MJ use. There is mixed evidence for how MJ use affects basal cortisol levels and the CAR. Future studies should consider MJ use characteristics, method of hormone collection, time when samples are collected, and environmental factors that may influence HPA axis activity in MJ users. By examining existing studies we provide one of the first reviews aimed at synthesizing the literature on HPA axis functioning in MJ users.

## Introduction

Marijuana (MJ) is the most commonly used illicit substance worldwide, with ~147 million past year users (1). Within the United States, MJ use has significantly increased during the past decades. Between 2001 and 2002, 4.1% of adults reported past year MJ use compared to 9.5% between 2012 and 2013, 30% of whom met criteria for cannabis use disorder (2). These increases in MJ use coincide with a time of MJ decriminalization, legalization, and changing attitudes regarding risk of MJ use (3). While multiple risk factors contribute to heavy MJ use, cumulative stress is one pathway that may be linked to chronic MJ use as individuals report using MJ to reduce stress (4).

Several studies indicate that the hypothalamic-pituitary-adrenal (HPA) axis, the major neuroendocrine system that responds to stress (5), is dysregulated in substance users (6, 7). In response to acute stress as well as substances of abuse, the HPA axis releases corticotropin-releasing hormone (CRH) from the hypothalamus, which promotes release of adrenocorticotropic hormone (ACTH) from the anterior pituitary gland, ultimately resulting in cortisol secretion from the adrenal cortex. However, chronic stress and heavy substance use can lead to allostatic load, HPA axis dysfunction, and adverse effects on stress responsivity (8, 9). Despite the worldwide prevalence of MJ use, little is known about HPA axis response in heavy MJ users. Understanding how MJ use affects HPA axis functioning in humans is critical to informing studies on the role of the neuroendocrine stress system in chronic MJ users and in individuals at risk for heavy MJ use.

The purpose of this review is to provide a descriptive overview of prior research on the effects of acute MJ administration on HPA axis activity, the impact of stress on HPA axis functioning in MJ users, and the role of chronic MJ use on basal cortisol levels and the cortisol awakening response (CAR). Additionally, we compare methodological differences among studies that may have contributed to discrepant findings, and comment on future directions for advancing research in this field. Articles for this mini review were included based on combinations of keywords searched on Pubmed, including “marijuana”, “HPA axis”, “cortisol”, “tetrahydrocannabinol (THC)”, and “endocannabinoid.” Titles and abstracts were reviewed for relevance to the topic on human MJ users. Additional articles were found through citations included within the manuscripts found using the keyword search.

## Effects of THC administration and acute marijuana use on HPA axis functioning in marijuana users

Several studies have investigated the effects of acute MJ administration on HPA axis response by examining ACTH and/or cortisol levels in a laboratory setting (Table 1). Cone et al. (11) found that MJ administration raised serum cortisol levels in MJ users compared to baseline. Similar results were reported by Kleinloog et al. (15), who reported that THC inhalation increased cortisol compared to baseline in infrequent MJ users. These findings were replicated in a resting state functional magnetic resonance imaging (fMRI) study, such that THC administration elevated cortisol levels compared to placebo in MJ users, but as there were no changes in hypothalamic connectivity observed, cortisol levels were not examined in relation to functional connectivity (16). In another study, De souse Fernades perna et al. (17) examined the effects of vaporized THC administration on cortisol response before and after an implicit association task displaying aggressive behavior, in which participants self-reported how aggressive they felt after viewing each image. MJ administration significantly elevated cortisol levels compared to placebo prior to aggression exposure. Cortisol levels were also higher after inhalation of MJ vs. unintoxicated baseline levels in an fMRI study examining the aphrodisiacal effects of MJ in MJ users. However, there were no differences in cortisol levels between MJ users with or without prior aphrodisiacal experiences, so its effect on brain activity was not examined further (18). Considering the lack of standardization of MJ administration, caution should be used when drawing conclusions based on these results. Overall, increases in cortisol after MJ administration may have both advantageous and disadvantageous effects. For example, as HPA axis activity mobilizes the body to face challenges, increased cortisol levels could be related to enhanced attention after acute MJ administration in heavy users (35), but could also be associated with impairments in other cognitive domains, such as working memory and inhibition (35), and increased anxiety (36). Thus, the increased cortisol response may be beneficial in certain contexts, but detrimental in others.

**Table 1:** Studies of HPA axis functioning in marijuana users.

**Study**	**Participants**	**Sample size**	**Age (mean ±SD)**	**Study design**	**ACTH or cortisol analysis**	**Main findings**
**ACUTE MJ ADMINISTRATION STUDIES**						
Benowitz et al. (10)	Regular MJ users (only male users)	*n* = 6	21–30	210 mg oral THC for 14 days,.15 U/kg IV insulin	Plasma cortisol	No acute effect of THC on cortisol; following THC treatment, insulin administration ↓ cortisol compared to pre-THC treatment levels
Cone et al. (11)	Frequent MJ users (only male users)	*n* = 4	33.75^a^	Inhaled 2 MJ cigarettes (2.8% THC), 1 MJ and one placebo cigarette, or 2 placebo cigarettes (one condition per day)	Plasma cortisol	↑ cortisol compared to baseline
Dax et al. (12)	Abstinent heavy and occasional MJ users (only male users)	*n* = 7 oral THC *n* = 6 inhaled THC *n* = 5 placebo	Age statistics not reported	10 mg oral THC (Marinol) or 18 mg/1.2 g inhaled MJ cigarette for 3 days (once on day 4)	Plasma ACTH and cortisol	No effect on cortisol for either method of administration
D' Souza et al. (13)	Abstinent MJ-dependent users and HC	*n* = 30 MJ users *n* = 22 HC	MJ = 24.8 ± 5.5 HC = 29 ± 11.6	0, 2.5, or 5 mg IV THC (one condition per day, test days separated by ≥1 week)	Plasma cortisol	Dose-dependent ↑ cortisol compared to placebo, blunted in frequent MJ users
Ranganathan et al. (14)	Abstinent MJ-dependent users and HC	*n* = 40 MJ users *n* = 36 HC	*MJ* = 28.28 ± 10.2 *HC* = 24.58 ± 4.9	Study 1: Placebo, 0.0357 mg/kg, 0.0714 mg/kg IV THC Study 2: placebo, 0.0286 mg/kg IV THC	Serum cortisol	Dose-dependent ↑ cortisol, blunted in frequent MJ users
Kleinloog et al. (15)	Mild MJ users (only male users)	*n* = 49 mild MJ users	18–45	2-, 4-, and 6 mg inhaled THC at 90 min intervals	Serum cortisol	↑ cortisol compared to baseline
Klumpers et al. (16)	Occasional MJ users	*n* = 12	22.17 ± 2.95	Day 1: 3 doses placebo Day 2: 2, 6, and 6 mg inhaled THC	Serum cortisol	↑ cortisol compared to placebo
De Sousa Fernandes Perna et al. (17)	Regular MJ users; heavy alcohol users; HC	*n* = 21 regular MJ users *n* = 20 alcohol users *n* = 20 HC	*MJ* = 21.9 ± 2.2 Alcohol = 22.7 ± 2.4 *HC* = 22.9 ± 2.3	300 μg/kg vaporized MJ (12% THC), aggression implicit association task (IAT)	Serum cortisol	↑ cortisol compared to placebo in MJ users administered THC prior to IAT
Androvicova et al. (18)	Casual MJ users	*n* = 12 aphrodisiac *n* = 9 non-aphrodisiac	Aphrodisiac = 29.08 ± 5.37 Non-aphrodisiac = 23.78 ± 3.03	Inhaled socially relevant doses of personal MJ 30 min prior to study visit	Serum cortisol	↑ cortisol compared to baseline
Childs et al. (19)	occasional MJ users, ≤ 1 use per week	*n* = 14: 0 mg THC *n* = 15: 7.5 mg THC *n* = 13: 12.5mg THC	0 mg *THC* = 23.8 ± 1.4 7.5 mg *THC* = 23.2 ± 0.9 12.5 mg *THC* = 23.9 ± 1.3	0, 7.5, or 12.5 mg oral THC (Marinol), Trier Social Stress Task	Salivary cortisol	No effect of THC on pre- or post- TSST cortisol levels
**STRESS ADMINISTRATION STUDIES**						
Van Leeuwen et al. (20)	Lifetime abstainers; lifetime tobacco users; and lifetime MJ users; users were also classified as repeated or lifetime users only	*n* = 219 lifetime abstainers *n* = 168 lifetime tobacco users *n* = 204 lifetime MJ users	16.27 ± 0.73	Groningen Social Stress Test	Salivary cortisol	↓ cortisol in lifetime MJ users vs. lifetime abstainers or lifetime tobacco users; similar finding in repeated MJ users vs. lifetime MJ or tobacco only users
Mcrae-Clark et al. (21)	MJ-dependent	*n* = 87	MJ stress group = 25.5 ± 9.2 MJ no stress group = 26.2 ± 8.0	Trier Social Stress Task; MJ cues	Plasma ACTH and cortisol	↑ ACTH and cortisol in stress group; ↑ cortisol in response to neutral vs. MJ cues
Somaini et al. (22)	Active MJ-dependent; abstinent MJ-dependent; HC	*n* = 14 MJ-dependent *n* = 14 abstinent MJ-dependent *n* = 14 HC	*MJ* = 24.1 ± 2.7 *HC* = 25.4 ± 3.6	Unpleasant and neutral pictures from IAPS	Plasma ACTH and cortisol	Active MJ-dependent ↑ basal ACTH and cortisol vs. other groups, but smallest ↑ in ACTH and cortisol after viewing unpleasant images
Fox et al. (23)	Treatment-seeking MJ, alcohol, cocaine dependent; alcohol/cocaine dependent; social drinking HC	*n* = 30 MJ, alcohol, cocaine- dependent *n* = 29 alcohol, cocaine- dependent *n* = 26 social drinking HC	MJ/alcohol/cocaine = 33.7 ± 6.9 alcohol/cocaine = 37.1 ± 6.4 *HC* = 28.1 ± 1.4	Guided imagery (stress, alcohol/cocaine cue, relaxing)	Plasma ACTH and cortisol	MJ-dependent polysubstance users ↑ ACTH and cortisol to stress vs. relaxing imagery; effect not seen in other groups
Tull et al. (24)	MJ-dependent PTSD; MJ-dependent no PTSD, PTSD only; no PTSD/no MJ-dependence	*n* = 18 PTSD/MJ *n* = 32 MJ no PTSD *n* = 27 PTSD only *n* = 91 no PTSD/no MJ	34.32 ± 10.1	Trauma cues	Salivary cortisol	No effect of trauma cues on cortisol
Cuttler et al. (25)	Daily or near-daily MJ users; HC	*n* = 40 daily MJ users *n* = 42 HC	MJ users in stress condition = 26.05 ± 1.44 MJ users in no stress condition = 25.14 ± 1.86 HC in stress condition = 26.95 ± 2.23 HC in no stress condition = 25.24 ± 1.19^b^	Maastricht Acute Stress Test	Salivary cortisol	↓ cortisol in daily MJ users vs. HC
Nusbaum et al. (26)	Daily or near-daily MJ users; HC	*n* = 39 daily MJ users *n* = 40 HC	MJ users in stress condition = 25.85 ± 6.19 MJ users in no stress condition = 25.35 ± 8.71 HC in stress condition = 27.25 ± 10.4 HC in no stress condition = 25.25 ± 5.57	Maastricht Acute Stress Test	Salivary cortisol	↓ cortisol in daily MJ users vs. HC
Chao et al. (27)	Non-treatment seeking daily MJ users with and without trauma exposure	*n* = 125	Age range 18–50 (more detailed demographics of the six subgroups in table of manuscript)	Trier Social Stress Task	Salivary cortisol	↑ cortisol before, during, after TSST in daily MJ users with trauma exposure vs. daily MJ users without trauma exposure
**BASAL CORTISOL STUDIES**^e^						
Block et al. (28)	Frequent, moderate, infrequent, or non-Users of MJ	*n* = 27 frequent MJ users *n* = 18 moderate MJ users *n* = 30 infrequent MJ users *n* = 74 non-users	23.5 ± 0.4^b^, more detailed demographics divided by user group and sex in table of manuscript	Morning or afternoon blood draw	Serum cortisol	No difference between groups
King et al. (29)	Daily or near daily MJ users; HC	*n* = 30 MJ users *n* = 30 HC	M MJ users = 21 F MJ users = 22.5 M *HC* = 23 F *HC* = 24.5^d^	Morning saliva collection	Salivary cortisol	↑ cortisol in MJ group compared to HC
Cloak et al. (30)	Heavy MJ users; light MJ users; HC	*n* = 43 heavy MJ users *n* = 37 light MJ users *n* = 42 HC	Heavy MJ users = 19.4 ± 0.3 Light MJ users = 19.1 ± 0.4 HC = 18.3 ± 0.4^c^	Late morning or afternoon saliva collection^f^	Salivary cortisol	No difference between groups
Carol et al. (31)	UHR youth with current MJ use; UHR youth without current MJ use; HC	*n* = 17 UHR with MJ use *n* = 26 UHR without MJ use *n* = 29 HC	UHR with MJ use = 19.59 ± 0.87 UHR without MJ use = 18.46 ± 1.92 *HC* = 17.34 ± 2.82	Three saliva samples every 60 min between 8:45 a.m.−2 p.m.	Salivary cortisol	↑ cortisol in MJ group compared to HC
Lisano et al. (32)	Physically active regular MJ users; physical active HC (males only)	*n* = 12 regular MJ users *n* = 12 HC	*MJ* = 23.33 ± 4.14 *HC* = 24.08 ± 5.5	Blood samples collected between 7 and 9 a.m.	Serum cortisol	No difference between groups
**CORTISOL AWAKENING RESPONSE STUDIES**						
Huizink et al. (33)	Early (9–12 years old), late (13–14 years old), and non-users of MJ	*n* = 59 late MJ users *n* = 44 early MJ users *n* = 1338 non-users	Cortisol collected between ages 10 and 12, age breakdown for groups not reported	Saliva collected at awakening, 30 min later, and 8 pm	Salivary cortisol	↓ cortisol 30 min post-awakening in early MJ users vs. late MJ users; MJ users ↑ evening cortisol vs. non-users
Montelone et al. (34)	SCZ with MJ use prior to psychotic symptoms; SCZ with no MJ use prior to psychotic symptoms; HC	*n* = 16 SCZ with MJ use *n* = 12 SCZ without MJ use *n* = 15 HC	SCZ with MJ use = 39.1 ± 7.2 SCZ without MJ use = 43.6 ± 7.3 *HC* = 37.6 ± 6.9^c^	Saliva collected at awakening, and 15, 30, and 60 min later	Salivary cortisol	↑ baseline cortisol in SCZ with MJ use vs. HC; ↓ CAR in SCZ with MJ use vs. HC

*ACTH, adrenocorticotropic hormone; CAR, cortisol awakening response; HC, healthy controls; IV, intravenous; MJ, marijuana; PTSD, post-traumatic stress disorder; SCZ, schizophrenia; THC, tetrahydrocannabinol; TSST, Trier Social Stress Task; UHR, ultra-high risk; ↑, increase/increased/greater; ↓, decrease/decreased/less*.

a *Standard deviation not reported*.

b *Mean ± standard error reported*.

c *Unclear if standard deviation or standard error reported*.

d *Medians reported*.

e *As there was no specific drug administration or acute stress manipulation, study design refers to time of day for blood or saliva samples to measure basal cortisol levels*.

f *A subset of participants completed the TSST and computerized neuropsychological battery and saliva was also collected before and after these tests, but no effects were found*.

In other studies, cortisol levels were compared between abstinent MJ-dependent individuals and non-users following intravenous THC administration. MJ-dependent individuals exhibited a blunted cortisol increase after THC administration compared to non-users (13, 14). Preclinical research has linked this blunted cortisol response to MJ tolerance (37), while other research suggests differences in MJ response may be influenced by genetics (38).

Some studies have found no significant effect of acute MJ administration on cortisol levels. In a study conducted by Benowitz et al. (10), the effects of insulin-induced hypoglycemia in MJ users were examined. Participants were given insulin prior to and after oral THC was administered. No difference in cortisol was observed between baseline and post-THC treatment but insulin administration decreased cortisol compared to pre-THC treatment levels. In a study by Dax et al. (12) abstinent MJ users were administered oral or inhaled THC. No differences were observed in ACTH or cortisol between baseline and post-treatment levels. Small sample sizes may have resulted in the lack of significant findings in these studies. Childs et al. (19) also found no relationship between acute administration of oral THC and cortisol response. The authors suggest their lack of findings could have been the result of collection of salivary rather than serum cortisol, the latter possibly being a more sensitive measure of cortisol. Moreover, in studies that found no effect of MJ on cortisol levels, greater time elapsed between MJ administration and cortisol assessment. It is possible the acute effects of MJ on cortisol could have diminished before cortisol assessment. Since studies that found an association between acute MJ administration and HPA axis response collected cortisol samples closer to the time of acute MJ use, it may be necessary for future studies to measure cortisol within 2 h following MJ administration. In sum, the majority of research examining acute MJ administration on cortisol reactivity has indicated MJ significantly increases cortisol, and some studies report that abstinent MJ-dependent users show a blunted increase in cortisol relative to non-users.

## Effects of stress on HPA axis functioning in marijuana users

A number of studies in MJ users have examined the effects of acute stressors on HPA axis activity (Table 1). A study by Somaini et al. (22) presented neutral and unpleasant images to MJ-dependent individuals, abstinent MJ-dependent individuals, and healthy controls. Interestingly, active MJ users had generally high basal stress hormone levels but reduced responsivity of the HPA axis, potentially due to dysregulation of the stress system by MJ use. These findings are in contrast to another study in which MJ-dependent polysubstance users had significantly higher levels of plasma cortisol and ACTH following exposure to stress imagery relative to relaxing imagery, a finding not present in non-MJ-dependent polysubstance users or social drinkers (23). Since participants in this study were abstinent treatment-seeking polysubstance users, the elevated cortisol and ACTH levels could reflect a “rebound” upregulation of the HPA axis following abstinence (23).

Two recent studies examined salivary cortisol in chronic adult MJ users using the Maastricht Acute Stress Test, which includes both physiological stress (placing hand in ice water) and psychosocial stress (solving math problems). The acute stress manipulation resulted in blunted cortisol response in the daily MJ users compared to healthy controls (25, 26). For individuals who may be characterized by an overactive HPA axis, a reduction in cortisol activity may be beneficial. Alternatively, cortisol release usually serves to motivate adaptive responses during stressful situations and a blunted response could impair one's ability to act appropriately (8, 25). In particular, a blunted cortisol response to psychosocial stress has been associated with anxiety and depression in women (39), suggesting female MJ users may be at increased risk for anxiety and depression symptoms. Similar findings were reported in a study with a large sample (*N* = 591) of adolescent MJ users who had lifetime or repeated MJ use (20). The authors reported lower salivary cortisol levels during the Groningen Social Stress Task (involving both a speech and math problems) in adolescents who had ever used MJ relative to non-users or participants who reported lifetime tobacco use. This finding was also seen when the authors compared adolescents who used MJ at least five times in the past year with lifetime users of MJ or tobacco. These results were interpreted as a reduction in HPA axis response in adolescents who are at risk for using MJ repeatedly, possibly to stimulate their HPA axis response. Finally, another study that utilized the Trier Social Stress Task found that stress increased plasma ACTH and cortisol levels in MJ-dependent participants (21). However, in response to MJ cues, cortisol levels were significantly lower in MJ-dependent participants than in response to neutral cues. As the purpose of this study was to examine the effects of stress and drug cues on physiological reactivity in MJ-dependent individuals, no control group was included.

Other types of stress, such as previous trauma exposure, which may influence cortisol response in MJ users, was examined in a study of non-treatment seeking daily MJ users (27). Daily MJ users who experienced trauma had higher overall cortisol levels before, during, and after the Trier Social Stress Task than those who had never experienced trauma. However, as there was no control group of non-using participants in this study, it is uncertain whether the effects of trauma on cortisol reactivity would be similar to or different from the daily MJ users. Contrary to the findings of this study, Tull et al. (24) found no effects on cortisol reactivity in participants with or without post-traumatic stress disorder who were either MJ-dependent or non-dependent, even though MJ-dependent participants reported less subjective emotional reactivity in response to trauma cues.

Taken together, the findings to date suggest that stress exposure in adult heavy MJ users (22, 25, 26), or adolescents at risk for heavy MJ use (20) is mostly related to blunted reactivity of the HPA axis. This could suggest both dysregulation as result of MJ use or increased vulnerability toward frequent MJ use as individuals may engage in MJ use to increase responsivity of an underactive HPA axis. As there is currently limited research in this area, future studies should carefully consider the following variables, which could impact study findings: method of obtaining cortisol sample [plasma: (21–23) vs. saliva: (20, 24, 25, 27)], time of day of cortisol measurement, duration of time between stress administration and cortisol measurement, MJ use criteria (frequency of use, MJ-dependent or non-dependent sample, treatment seekers vs. non-treatment seekers), and co-occurring mental health conditions, such as previous trauma exposure (24, 27) or psychopathology (23).

## Marijuana use and basal HPA axis activity

Studies have measured cortisol levels in frequent MJ users and non-users to determine whether the groups differ in basal cortisol levels, and findings suggest MJ has either no effect or increases basal cortisol (Table 1). Block et al. (28) found that there was no difference in serum cortisol levels between frequent, moderate, and infrequent MJ users and controls. However, only one blood sample was taken and time of day for blood draws varied among participants. There was also no difference in serum cortisol response in physically active MJ-using adults compared to non-using controls, suggesting that heavy MJ use may not affect stress hormone levels in individuals with high levels of physical activity (32). Since previous studies report that MJ may be used to reduce stress and anxiety symptoms (4), Cloak et al. (30) examined the relationship between MJ use, anxiety symptoms, and cortisol levels in adolescent and young adult heavy, light, and non-MJ users. There was no effect on mid-day salivary cortisol despite greater MJ use being associated with more anxiety symptoms, indicating a disconnect between psychological, and physiological stress reactivity.

Contrary to the findings above, an fMRI study examining psychomotor function found that chronic MJ users had higher levels of salivary cortisol compared with controls and greater superior frontal gyrus (SFG) but reduced visuomotor activity relative to controls (29). The authors propose that this increased cortisol in MJ users may impair visuomotor function during psychomotor tasks, resulting in greater reliance on brain regions involved in attention and motor planning, such as the SFG. A recent study of adolescents at ultra-high risk for schizophrenia reported that youth who used MJ in the past month had higher levels of salivary cortisol than healthy controls, suggesting a potential link between risk for psychosis and HPA axis functioning (31). Previous research indicates high basal cortisol levels are associated with hypertension and obesity (40), as well as hippocampal atrophy and memory impairment in aging populations (41). The potential effect of frequent MJ use on basal cortisol levels requires further investigation to clarify inconsistencies in the literature. Variations in participant characteristics, MJ use parameters, and method of cortisol assessment may have contributed to the inconsistent findings.

## Cortisol awakening response in marijuana users

Cortisol levels exhibit diurnal variation, such that levels rise during the morning hours, peak 30 min after awakening, and are lowest in the evening. This increase of cortisol in the morning, known as the CAR is believed to be a reliable marker for individual differences in HPA axis activity (42). Studies have reported that the CAR is influenced by substance use, such as heavy alcohol use (43, 44). Surprisingly, little is known about the CAR in MJ users (Table 1). To our knowledge, only one study to date has examined diurnal cortisol response in MJ users, and found blunted levels of cortisol 30 min after awakening in a large sample of children (10–12 years old) who began using MJ during early adolescence (9–12 years old) relative to those who initiated use in later adolescence (13–14 years old) (33). These findings may indicate that blunted cortisol response could be a risk factor for initiating MJ use. The study also found that participants who initiated MJ use regardless of age at first use, had higher levels of evening cortisol relative to non-users. The authors believed this finding may be explained more by environmental influences on cortisol levels in the evening, such as ongoing stressful events rather than genetic vulnerability toward MJ use. Similar findings were reported in another study, albeit in a sample of participants diagnosed with schizophrenia who were also MJ users (34). These participants had higher baseline levels of cortisol, but a flattened CAR relative to healthy controls. These findings may indicate that MJ use in schizophrenics contributes to dysregulation of the HPA axis, although it is possible that blunted CAR in MJ-using schizophrenics predated and increased their vulnerability toward substance use. Given the lack of research investigating the CAR in MJ users, significant work is needed to characterize how MJ use affects the CAR and whether dysregulation of HPA axis functioning is a risk factor for and/or further drives MJ use.

## Conclusions and future directions

The purpose of the current mini review is to highlight and integrate the existing, albeit limited literature on the effects of MJ use and stress on HPA axis functioning in adult MJ users and youth at risk for heavy MJ use. Understanding these findings comes at an important time when MJ decriminalization and legalization has made MJ increasingly available, while the perceived risk of MJ use has declined (3). Preclinical research has indicated that cannabinoids affect functioning of the HPA axis [for review, see Steiner and Wotjak (45)], and it appears that the current findings suggest that overall, acute MJ administration elevates cortisol levels, but to a smaller degree in MJ-dependent users. Further, acute stress exposure in heavy MJ users also appears to largely blunt cortisol reactivity. The findings on basal cortisol levels are mixed, likely due to diurnal fluctuations of cortisol and because cortisol is sensitive to changes in daily stress. These findings suggest that MJ use may dysregulate normal functioning of the HPA axis, perhaps as individuals develop tolerance to MJ, which could further drive MJ use. An alternative explanation is that individuals at risk for MJ use seek out MJ to stimulate an underactive HPA axis. Further research, including longitudinal studies of MJ users to examine long-term effects on HPA axis functioning are needed. While there is a growing literature on the effects of MJ use on brain structure and functioning in humans (46, 47), few studies have measured HPA axis activity in neuroimaging studies of MJ users, an important avenue for future research. This could provide information on how biological markers related to stress reactivity are associated with neurocognition in MJ users. Additionally, to our knowledge only two studies have examined the CAR in MJ-using participants, thus necessitating significant work in understanding how MJ use affects diurnal HPA axis rhythms. Work is currently underway in our laboratory to examine the effects of adult heavy MJ use on the CAR.

## Author contributions

All authors contributed to the article search for this review. AC and JD-B wrote the Introduction, SL wrote the section on acute THC and MJ effects on HPA axis functioning, and AC wrote the sections on stress, basal cortisol, cortisol awakening response, and conclusions and future directions. AC and SL created the table. AC edited and finalized the manuscript.

### Conflict of interest statement

The authors declare that the research was conducted in the absence of any commercial or financial relationships that could be construed as a potential conflict of interest.
